# Theory of Mind and Concealing vs. Forthcoming Communication in Adolescence

**DOI:** 10.3389/fpsyg.2022.866964

**Published:** 2022-04-14

**Authors:** Jennifer Lavoie, Victoria Talwar

**Affiliations:** ^1^Moray House School of Education and Sport, University of Edinburgh, Edinburgh, United Kingdom; ^2^Department of Educational and Counselling Psychology, McGill University, Montreal, QC, Canada

**Keywords:** early adolescence, concealment, theory of mind, adolescent development, relationships

## Abstract

Concealing information requires that adolescents manage the information that they share, which requires cognitive skills, for example, theory of mind (ToM). This study explored motivations for concealment that early adolescents (*N* = 90, *M* = 12.81 years, *SD* = 5.10 months, range 12–14 years, and 58% female) endorsed concealing or disclosing to friends and parents, in relation to their theory of mind. We found that adolescents broadly endorsed disclosure to both parents and friends, even when it might mean they would face consequences, be impolite (by not protecting another’s feelings), or face negative identity-related emotions. We found that ToM ability was associated with a tendency to endorse being forthcoming and sharing information with both friends and parents. These findings provide new insight into how the relation between ToM and concealment may change with age, specifically how in early adolescence it may foster open communication rather than concealment as is the case in early and middle childhood.

## Introduction

Secrets, that is, information that is consciously concealed from at least one person (Bok, 1983; Kelly, 2002), are a component of everyday life. They are shared and concealed on a daily basis by children and adults alike. Determining who to tell a secret to, or who to keep a secret from, can contribute to a sense of personal autonomy or augment a shared group identity (Finkenauer et al., 2002). Because of this, secrets have had, and continue to have a positive and protective role in society. However, secrets can also be harmful. Secrets that are shared outside of an accepted group membership, or at an inopportune moment, or secrets that put a person at risk or in jeopardy can also negatively affect personal life (Hunter et al., 2011). These examples highlight the sometimes fine line between the positive and adaptive nature of secrecy and the negative side of secrecy.

For adolescents in particular, the decision to conceal information or disclose becomes particularly salient during a critical part of their development as they begin the process toward becoming autonomous from their parents (Darling et al., 2006). In fact, previous studies with adolescents have found that they are more likely to disclose secretive information to friends than parents (e.g., Villalobos Solís et al., 2015; Elsharnouby and Dost-Gözkan, 2020). When studied broadly, both boys and girls report similar levels of concealment (Finkenauer et al., 2005), but this may vary when the topic of concealment (or disclosure) is studied more closely. For example, in cases of disagreement, adolescent girls, more so than adolescent boys, are likely to disclose to parents (Darling et al., 2006). Thus, understanding how young adolescents perceive different types of information as secretive and how they would share or conceal such information can provide insight into how and when adolescents may use concealment vs. disclosure in emerging adolescence as young people begin the process of increasing in autonomy.

### Spectrum of Concealment Methods

There are many approaches that can be used to conceal information. Adolescents may choose to simply withhold information, and in doing so, use secrecy as a passive approach to conceal information. They may also choose to a more active approach to concealing information, through lying (Engels et al., 2006; Lavoie and Talwar, 2020) to present a false scenario. Conversely, they may be forthcoming and disclose the information requested (Smetana et al., 2006), and in doing so, not conceal information. Finally, they may also use a combination of these approaches and partially conceal information but be forthcoming about other information (Cumsille et al., 2010).

Of all of the above-mentioned approaches (secrecy, lying, disclosure, and partial disclosure), lying is considered to be the least desirable approach and can undermine trust in relationships (Engels et al., 2006). In contrast, disclosure can help to facilitate open communication in relationships and can build a healthy autonomy in the parent–adolescent relationship as parent–adolescent dyads navigate difficult conversations successfully (Collins and Luebker, 1994). In terms of relationships with friends, sharing secrets can be considered a form of social “currency” and build bonds of trust and friendship, creating a sense of shared group belonging (Lavoie et al., 2017b). Previous research studies have found that adolescents tend to disclose more to best friends than to parents (Frijns et al., 2013; Villalobos Solís et al., 2015; Elsharnouby and Dost-Gözkan, 2020; mothers, then fathers specifically; Elsharnouby and Dost-Gözkan, 2020). Thus, in both relationships—with parents and with friends—open disclosure of information can help to build and solidify the relationships in a healthy way, though it is possible that the types of information (i.e., the motives for concealment) that is shared with parents vs. friends may differ.

Theory of mind (ToM) may be associated with patterns of concealment and disclosure, given that multiple previous studies have found an association between ToM and deception (Talwar and Lee, 2008; Evans et al., 2011; Ding et al., 2015; Talwar et al., 2017). In fact, a recent meta-analysis on the relation between ToM and lying indicates that ToM is associated with all aspects of lying, with a stronger relation to lie maintenance (i.e., ability to lie successfully without leaking incriminating information) than to spontaneous lying (Lee and Imuta, 2021). Further, both ToM and working memory differentiate between young children’s concealment and disclosure behavior, with lower ToM and working memory predicting disclosure of a secret to a parent (Lavoie and Talwar, 2020). Thus, there is plausible reason to think that ToM may be differentially associated with forms of concealment and disclosure more broadly. Our study focused on the types of concealment methods that early adolescents endorse using, in relation to their cognitive and affective ToM, as discussed further below.

### Theory of Mind and Concealment

Concealing information requires that adolescents manage the information that they share (Smetana et al., 2006), which requires cognitive skills, for example, inhibitory control and working memory (Gordon et al., 2014) to determine and remember what information can be shared to avoid inadvertently revealing the hidden information. One key cognitive skill that supports effective concealment is ToM. ToM is the ability to understand and infer another individual’s mental state (Premack and Woodruff, 1978), which involves putting oneself in another’s perspective to determine knowledge that the individual would have, as well as gaging their mental state.

Research on children’s lie-telling has reported ToM as a critical ability for the emergence of lying (Leduc et al., 2017) and for its development (Talwar et al., 2007; Talwar and Lee, 2008; Lavoie et al., 2017a). Children who have more advanced ToM skills are better at maintaining and giving more elaborate lies (Talwar et al., 2007; Talwar and Lee, 2008; Lavoie and Talwar, 2020). However, recent evidence suggests that the relation between ToM and the types of lies children tell may change with age and may not be linear. For example, Lavoie et al. (2017a) found that although younger children’s lie-telling was related to better ToM, emerging adolescents with higher ToM were more likely to only tell occasional lies that were more prosocial in nature. In this case, their findings suggest that the type of lie, or the motive for telling a lie, is a key differentiating factor between ToM and concealment. Lavoie and colleagues measured seven motives for lying: to avoid consequences, for an instrumental purpose, to blame another person, to protect the self from embarrassment, and to protect another, polite lies, and lies told out of play. The authors gathered these seven categories from previous literature on the motives for lying (e.g., DePaulo et al., 2004). Thus, the results from these findings suggest that the types of patterns of deception that children and early adolescents use in their daily lives are associated with ToM ability (Lavoie et al., 2017a), and this relation may also extend to the motivations for concealment that early adolescents endorse concealing vs. disclosing to parents and friends, as we examined in this study.

### Cognitive vs. Affective ToM

Within the literature on ToM, a distinction has been made between cognitive and affective ToM (otherwise known as “cold” and “hot” aspects of ToM; Brothers and Ring, 1992). Affective ToM is the ability to recognize and infer others’ emotional states and can be thought of as an empathic understanding, whereas cognitive ToM involves the ability to make inferences about others’ thoughts and beliefs (Shamay-Tsoory and Aharon-Peretz, 2007; see also Geraci et al., 2010 and Geraci and Cantagallo, 2011 in support of a dissociation between emotional and cognitive aspects of ToM following a brain injury or schizophrenia). As children approach adolescence, they use their overall ToM ability to navigate their relationships with themselves and others (Devine and Hughes, 2013), but they may also use cognitive vs. affective ToM differently in social relationships. For example, a greater social perceptual ability (affective ToM) may be strongly associated with increased disclosure out of a desire to build trusting relationships through open dialogue, whereas a greater social cognitive ability (cognitive ToM) may be more so associated with methods of concealment out of the confidence that the information recipient may never become aware of concealment or potential deception. This hypothesis presents how early adolescents may use these two ToM abilities differently in determining what to share vs. disclose in social relationships, however, this has not yet been tested.

### Current Study

The purpose of this study was to (1) examine the types of secrets that adolescents endorse sharing vs. concealing to friends vs. parents; (2) explore whether different motives for concealment to parents vs. friends, having an overall high tendency for concealment, or disclosure to parents about specific domain issues were associated with ToM ability, taking into consideration gender.

We expected that the majority of early adolescents would endorse some concealment at least some of the time, based on past research that suggests that the majority of adolescents do endorse keeping secrets from parents on at least some types of issues (Darling et al., 2006). We also anticipated that we might see gender differences, given that boys would endorse more concealment methods for topics relating to more personal matters (Smetana et al., 2006; e.g., embarrassing information and self-incriminating information). We further anticipated that adolescents would be more likely to endorse methods of concealment to parents than peers.

Regarding theory of mind, we anticipated that higher theory of mind, specifically the ability to infer mental states (i.e., affective ToM), would be associated with higher endorsement of disclosure, potentially as a way to facilitate relationships through open communication, dialogue, and to build trust in relationships (Koenig and Harris, 2005). We expected conversely that higher theory of mind through the ability to follow third-party information transfer (false location tasks; i.e., cognitive ToM) would be associated with higher endorsement of concealment methods because participants might feel more confident that the information recipients may never become aware of concealed information (if they are skilled at keeping track of what was shared vs. not shared).

## Materials and Methods

### Participants

Participants were 90 early adolescents (*M* = 12.81 years, *SD* = 5.10 months, range 12–14 years, and 58% female) in a mid-large metropolitan city in North America. Participants’ parents provided written consent and commonly reported an educational background of a university-level education (48%), college diploma (22%), or post-graduate education (11%), as well as a household income level of $90,000 or greater (69%). Participants provided written consent and verbal assent to take part in the study. Information on ethnicity was not collected, but participants were recruited from inner-city metropolitan communities with a high level of diversity.

### Measures

#### ToM False Location (Cognitive ToM)

Participants completed two second-order false location story tasks, stories adapted for age appropriateness from Hogrefe et al. (1986) and Sullivan et al. (1994). For each story, participants read a short vignette and answered four questions that assessed their understanding of the story. There were two control questions per story, which participants must have answered correctly to be eligible to receive a point for the target questions, two target questions that assessed participants’ ability to follow the flow of information from the perspective of two story characters to determine how the characters would most likely respond, and one “why” question that asked participants to explain one of their target responses. Each target question was scored out of a possible one point, and the “why” question was scored out of two possible points (no points for an incorrect or not relevant response, one point a behavioral justification, two points for a psychological mental context understanding response), for a total of eight points between the two stories. There were no ceiling effects.

#### ToM Mental States (Affective ToM)

Participants completed the Reading the Mind in the Eyes Test—Third Edition (Baron-Cohen et al., 2001) to measure their ability to infer complex mental states based on an image of a person’s face. Participants looked at 36 different facial emotions and selected the best response from a selection of four possible responses. One point was awarded for each correct response, for a total out of 36 points.

#### Motivations to Conceal Vignettes

Participants read 12 short vignettes (provided in Supplemental Material) that outlined a situation in which the story character was in a position to have a plausible motivation to conceal information from a friend or parent. Three motivations were used: to avoid consequences for one’s actions, to protect another person’s feelings from the blunt truth, and to protect oneself from embarrassment or identity-related negative emotions (DePaulo and Kashy, 1998; DePaulo et al., 2004; Talwar and Crossman, 2011; Lavoie et al., 2017a). We selected these three motivations because they touch on the spectrum of “antisocial” motives (avoid consequences) and “prosocial” motives (protect another person), as well as relatively “neutral” motive (perhaps tending toward antisocial depending on the context; protect oneself from negative identity-related emotions).

At the end of each vignette, the participant was asked whether the story child would likely respond openly to the recipient, keep the information a secret, or lie to cover up the information, to reflect a “spectrum” of concealment responses, ranging from forthcoming disclosure (scored as “1”), passive concealment (scored as “2”), or active concealment (scored as “3”; e.g., Lavoie and Talwar, 2020). A mean score was generated for all responses to a friend disclosure recipient (six total responses), as well as for a parent disclosure recipient (six total responses). A mean score was also generated for scenarios to protect another (four total responses), to protect self (four total responses), and to avoid consequences (four total responses).

#### Self-Concealment Scale

The Self-Concealment Scale (Larson and Chastain, 1990) was used to measure participants’ general tendency to keep information secretive or private from others. Ten items were scored on a five-point Likert scale from *“strongly disagree”* to *“strongly agree,”* Cronbach’s *α* = 0.85. A mean score was calculated for the scale as a whole.

#### Youth Disclosure Scale

Participants completed the Youth Disclosure Scale (Smetana et al., 2006) to measure their forthcomingness to parents in different domains of their lives. There were 12 items across three domains (personal issues, five items, Cronbach’s *α* = 0.86; peer issues, three items, Cronbach’s *α* = 0.66; and schoolwork issues, four items, Cronbach’s *α* = 0.52) that were scored on a five-point Likert scale from *“never tell”* to *“always tell.”* A mean score was calculated for each of the three domains.

### Procedure

Participants visited a child research laboratory with a parent/guardian and signed consent (parents and participants) and provided verbal assent before being invited to complete a series of pencil-and-paper questionnaires in a separate quiet room. Participants were told they could pass or skip any item they did not feel comfortable answering. All study materials and procedure were approved by the relevant university research ethics committee following the standards set out by the American Psychological Association.

## Results

### Preliminary Analyses

**Figure 1:**
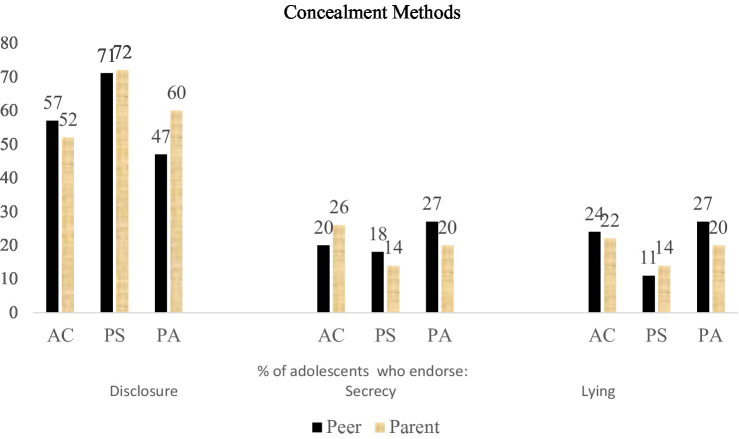
Concealment methods endorsed (in %), according to motive for concealment and information receiver. AC, avoid consequences; PS, protect self; and PA, protect another.

As a first step, we conducted preliminary analyses to determine whether there were significant relations between the main concealment and disclosure measures, subdivided by gender (Table 1). A visual breakdown of concealment responses is provided in Figure 1. We found that correlations between disclosure topics (i.e., Youth Disclosure Scale) were stronger in the male sample than the female sample, and that overall there were associations between the concealment and disclosure measures, ranging from a non-significant correlation between disclosure to parents about school work-related topics and endorsement of concealment from friends across the three motivations to conceal (among the male participant sample), *p* = 0.008, and a strong correlation between endorsement of disclosure to parents about school work-related issues and about peer-related issues, *p* < 0.01 (again among the male participant sample).

**Table 1:** Correlations between disclosure and concealment variables.

	1	2	3	4	5	6
1. Disclosure to parents—schoolwork	-	0.421^**^	0.410^**^	−0.059	0.197	0.031
2. Disclosure to parents—peer issues	0.605^**^	-	0.537^**^	−0.213	0.055	−0.247
3. Disclosure to parents—personal issues	0.449^**^	0.720^**^	-	−0.355^*^	0.015	−0.129
4. Self-concealment	0.183	−0.078	−0.080	-	0.384^**^	0.282
5. Concealment—friend	0.008	−0.058	−0.064	0.130	-	0.726^**^
6. Concealment—parent	−0.134	−0.264	−0.178	0.174	0.602^**^	-

Correlations above the diagonal are with female participants, below the diagonal are with male participants.

* *p* < 0.05;

** *p* < 0.01.

### ToM Abilities

Next, we examined whether ToM was associated with concealment and disclosure. We used linear regression modeling for each of our outcomes, given the relative statistical distinction (i.e., there were no strong correlations between the outcomes, which suggests that they each measure a distinct enough aspect of concealment to merit separate analyses rather than a mathematically combined construct).

#### Motivations to Conceal Vignettes

We conducted linear regressions with each of the concealment outcomes (concealment from parent, concealment from friend, and concealment by type: protect another, protect self, and avoid consequences). Gender, ToM false location score, and ToM mental states score were the predictors for each model.

The model predicting concealment from friends was significant, *F*(3, 86) = 3.78, *p* = 0.014, *R^2^* = 0.12, and explained approximately 12% of the variance in concealment from friends. ToM mental states score was a significant predictor of concealment measures from a friend, *b* = −0.03, *SE* = 0.01, and *p* = 0.014 [95% *CI* −0.05, −0.01]. With increasing ability to infer complex mental states, adolescents were more likely to be more forthcoming with their friends rather than using active concealment methods (see Table 2 for model information for all significant models).

**Table 2:** Model information for concealment and disclosure outcomes.

	*B*	SE	*t*	*p*	95% CI
*Concealment from friends*, *F*(3, 86) = 3.78, *p* = 0.014					
Intercept	2.25	0.31	7.24	0.000	1.63, 2.86
Gender	0.11	0.10	1.13	0.262	−0.08, 0.30
ToM false location	−0.03	0.03	−0.88	0.380	−0.09, 0.03
ToM mental states	−0.03	0.01	−2.51	0.014	−0.05, −0.01
*Concealment to protect the self*, *F*(3, 87) = 4.67, *p* = 0.005					
Intercept	2.39	0.30	8.00	0.000	1.80, 2.99
Gender	−0.02	0.09	−0.20	0.841	−0.20, 0.16
ToM false location	−0.06	0.03	−2.14	0.035	−0.12, −0.004
ToM mental states	−0.03	0.01	−2.40	0.019	−0.05, −0.004

We also found that the model predicting concealment measures to protect the self was also significant, *F*(3, 87) = 4.67, *p* = 0.005, *R^2^* = 0.14. Approximately 14% of the variance in adolescents’ concealment judgments about situations to protect the self were explained by the predictors. Both measures of ToM were significant predictors, ToM false location score, *b* = −0.06, *SE* = 0.03, *p* = 0.035 [95% *CI* −0.12, −0.004], and ToM mental states score, *b* = −0.03, *SE* = 0.01, *p* = 0.019 [95% *CI* −0.05, −0.004]. Children with higher ToM were more likely to be more forthcoming with friends and parents in scenarios in which self-protection were motivating factors to use concealment methods.

There were no significant predictors in concealment from parent [*F*(3, 86) = 1.20, *p* = 0.316, *R^2^* = 0.04], concealment to protect another [*F*(3, 86) = 0.93, *p* = 0.428, *R^2^* = 0.03], and concealment to avoid consequences, [*F*(3, 86) = 2.57, *p* = 0.059, *R^2^* = 0.09].

#### Self-Concealment

We next assessed the relation between ToM and general self-concealment to determine whether ToM might predict overall self-concealment, in terms of some adolescents being more secretive in general. We used gender, the ToM false location scores, and the ToM mental states scores as the predictors, and self-concealment score as the outcome. Neither gender nor ToM scores predicted self-concealment, and the model was not significant, *F*(3, 74) = 1.08, *p* = 0.363, *R^2^* = 0.04.

#### Youth Disclosure Scale

Finally, we assessed the relation between ToM and the topical areas that youth endorse disclosure of information to parents, which were personal issues, peer issues, and schoolwork issues. We used linear regression models to predict these outcomes, with gender, ToM false location scores, and ToM mental state scores as predictors. However, none of the models were significant; disclosure to parents about personal issues, *F*(3, 76) = 1.25, *p* = 0.296, *R^2^* = 0.05, disclosure to parents about peer issues, *F*(3, 73) = 0.614, *p* = 0.608, *R^2^* = 0.03, and disclosure to parents about schoolwork issues, *F*(3, 76) = 1.60, *p* = 0.197, *R^2^* = 0.06.

## General Discussion

Overall, we found that adolescents were most likely to endorse disclosure to parents and friends for each of the three motivations for concealment: to avoid consequences, to protect another, and to protect oneself from negative identity-related emotions. Adolescents were also most likely to endorse disclosure, in a broad sense, to friends more so than parents. Further, we found that theory of mind ability was associated with a tendency to endorse being forthcoming and sharing information with both friends and parents, which suggests that ToM may be a facilitator ability that helps foster honesty in close relationships by early adolescence, contrary to early and middle childhood as we discuss further below.

### Theory of Mind and Disclosure

A key finding that emerged from our study is the relation between higher ability to infer mental states and endorsing being more forthcoming with friends, rather than using active concealment methods. Prior studies have demonstrated that ToM is a necessary skill to be able to conceal in the first place (Peskin, 1992; Polak and Harris, 1999) and that ToM is associated with concealment and a better ability to conceal in early and middle childhood (e.g., Talwar and Lee, 2008; Ding et al., 2015; Fu et al., 2018; Lavoie and Talwar, 2020). However, our findings also suggest that, specifically for early adolescents, higher ToM is likely associated with higher disclosure in close personal relationships. We found this in our vignettes about a hypothetical child, but there is reason to suspect this may reflect adolescent’s own response tendencies. In other words, adolescents with this mentalizing ability may endorse disclosure with friends as a way of building and maintaining trust, as sharing secretive information can be a form of social currency to build bonds of friendship (Koenig and Harris, 2005).

This new finding also suggests that the relation between ToM and concealment is not linear across childhood; rather, as children enter adolescence their mentalizing abilities are used to regulate their information-sharing to prioritize building relationships of trust. In other words, mentalizing abilities support the initial development of concealment in childhood, but in the transition to adolescence, those with better mentalizing abilities may actually be more judicious in using concealment and may understand the benefits of open communication in their close relationships. This also aligns with previous findings that the deception patterns of young adolescents with high ToM were most likely to be infrequent and prosocial in nature, as a way of protecting relationships (Lavoie et al., 2017a). This protecting relationships is a type of prosocial behavior (i.e., protective behavior in defense of another, Geraci and Franchin, 2021), and highlights that the relation between ToM and concealment likely changes throughout childhood in terms of how acceptable and adaptive it is to conceal information vs. disclose it. In this way, this study provides new evidence that a prosocial motivation may be a factor underlying the relation between ToM and concealment and disclosure in early adolescence.

We also found that a higher ability to infer mental states as well as a higher ability to process false location information (broadly social cognition; Tager-Flusberg and Sullivan, 2000; Carlson et al., 2002) was associated with being more forthcoming to both friends and parents in situations where the adolescent would have a plausible motivation to protect themselves. This was somewhat surprising given that our original hypotheses contrasted the relation between the two theory of mind measures and disclosures, specifically that ability to infer mental states would predict disclosure and ability to process false location information would predict concealment methods given that the speaker may have better semantic leakage control (i.e., not “leaking” incriminating information that suggests they are concealing or deceiving the information receiver; Talwar et al., 2007). At the same time, the findings of previous studies suggest that adolescents are least likely to endorse disclosing personal information (Smetana et al., 2006) and information that an individual may think would hurt themselves or another person (Afifi et al., 2005). In this way, our findings extend those of prior studies to indicate that, in fact, adolescents with high theory of mind ability are more likely to disclose to friends and parents, even when the information could make them feel ashamed or embarrassed.

### Concealment Methods According to Situation

We found that disclosure was more commonly endorsed than methods of concealment, a finding that was consistent across situations to avoid consequences, to protect the self, and to protect another. This is consistent with previous research that has found that disclosure is the most commonly endorsed communication strategy among adolescents, when considered holistically across situation types (Cumsille et al., 2010). Yet, this finding stands somewhat in contrast to the finding of Darling et al. (2006) who found that the majority of adolescents endorse concealment from parents at times.

Within the types of situations we tested, young adolescents were most likely to endorse sharing openly in situations where their motivation to conceal would be to protect themselves (e.g., from embarrassment or negative emotions), to both peers and parents. This suggests the young adolescents were comfortable sharing openly even though the subject matter could be considered sensitive, and may also imply they had healthy close relationships. Results from previous research have found that adolescents (aged approximately 14–18 years) are least likely to endorse disclosing personal issues to parents (in comparison with schoolwork or peer issues; Smetana et al., 2006), and together with our findings, suggests younger adolescents may be more forthcoming about personal matters than older adolescents. From the parents’ perspective, parents of adolescents feel their child is less obliged to disclose personal information as the child gets older (Smetana et al., 2006); thus, our findings suggest there may be developmental differences in the types of information young people are comfortable sharing between early adolescence and later adolescence.

Adolescents in our sample indicated similarities between what they would disclose to a peer or a parent (as seen in Figure 1), but an overall preference of disclosure to peers. A handful of previous studies have found that adolescents tend to prefer sharing information with peers over parents (e.g., Villalobos Solís et al., 2015; Elsharnouby and Dost-Gözkan, 2020), and there is also some evidence to suggest that adolescents may feel more comfortable disclosing sensitive information (regarding sexual abuse, Priebe and Svedin, 2008; regarding aversive exchanges, Vernberg et al., 1995) to peers than authority figures. Although our findings align with previous findings, the differences that we found between disclosure to peers vs. parents were not particularly striking. It could be that the situations we tested were relatively innocuous and, as such, any peer vs. parent differences were relatively minor. It may also be that early adolescents do not yet have a strong distinction or preference between disclosing information to parents vs. peers, which should be tested in future research.

### Implications, Limitations, and Future Directions

The findings of this study have implications for individuals who work and interact with early adolescents in various settings and who may be looking to facilitate environments of open communication where adolescents feel comfortable sharing sensitive information, for example, parents, educators, and psychologists. Perhaps one key area for application is that adolescents’ cognitive ability, through their theory of mind, is associated with their disclosure vs concealment preferences. For adolescents with high social cognition (i.e., high levels of awareness of the people and their feelings in their environment), being cognizant of how sensitive information is received, through monitoring verbal and non-verbal reactions to adolescent’s disclosures, is one way through which a welcoming environment for disclosures can be created to facilitate open and honest communication.

There are also several limitations to the current study. First, previous studies have found that the information that adolescents disclose to parents differs between mothers and father (e.g., Smetana et al., 2006). We did not differentiate between information receivers, and consequently, our results may be a more global indicator of disclosure to parental figures. For future studies, considering both mothers and fathers and information receivers would help to further understand the instances in which adolescents are more likely to disclose information. Second, measures of relational quality and trust (e.g., Rotenberg, 1995) for peer groups and parents were not included, but would help to further tease apart the findings of our study. Third, distinctions between children’s actual concealment behavior and their endorsements of concealment vs. disclosure can be teased apart in future studies to assess overlap between the two. Finally, our measure of affective ToM required only the ability to attribute a mental state, and not to infer why such a mental state occurred, and as such future studies may benefit from including additional questions when measuring affective ToM to also capture both first- and second-order cognitive ToM reasoning.

## Conclusion

In conclusion, we found that disclosure was endorsed to both parents and friends when the motivation was to protect another, rather than using concealment methods that might protect the other person’s feelings. Friends tended to be the preferred recipients overall for information for which there is a motivation to conceal, specifically to avoid consequences or to protect oneself from negative identity-related emotions, but overall this difference was not particularly strong. Of note, higher theory of mind was associated with a greater willingness to disclose to both friends and parents. These findings are a key to providing new insight into how the relation between ToM and concealment changes during adolescence, toward supporting disclosure, rather than concealment as is the case in early and middle childhood. Finally, these findings suggest that a prosocial motivation may be an underlying factor to the relation between ToM and concealment and disclosure in early adolescence.

## Data Availability Statement

The raw data supporting the conclusions of this article will be made available by the authors, without undue reservation.

## Ethics Statement

The studies involving human participants were reviewed and approved by McGill University Research Ethics Board. Written informed consent to participate in this study was provided by the participants’ legal guardian/next of kin.

## Author Contributions

JL and VT contributed to the conceptualization of the research questions, design of the study, and the manuscript preparation. All authors contributed to the article and approved the submitted version.

## Conflict of Interest

The authors declare that the research was conducted in the absence of any commercial or financial relationships that could be construed as a potential conflict of interest.

## Publisher’s Note

All claims expressed in this article are solely those of the authors and do not necessarily represent those of their affiliated organizations, or those of the publisher, the editors and the reviewers. Any product that may be evaluated in this article, or claim that may be made by its manufacturer, is not guaranteed or endorsed by the publisher.

## Supplementary Material

The Supplementary Material for this article can be found online at: https://www.frontiersin.org/articles/10.3389/fpsyg. 2022.866964/full#supplementary-material
