# Supplementary material for: Theory of Mind and Concealing vs. Forthcoming Communication in Adolescence
Source: Front Psychol. 2022 Apr 14;13:866964. doi: 10.3389/fpsyg.2022.866964 (PMC9046603; doi:10.3389/fpsyg.2022.866964)
Supplement: Supplementary file 1 [file Data_Sheet_1.docx]

Supplementary Material

Motivations to Conceal Vignettes

1. Sam’s teacher handed back their math tests in class. Sam had studied really hard and was expecting an A on the test, but when he got his test back, it was a B. Sam’s best friend, Ben, asked Sam what Sam got on the test. How is Sam most likely to respond to Ben?
2. When Sam got home, his parent asked him if he had received his grade on his math test. His parent knew that Sam had studied really hard and was expecting an A. How is Sam most likely to respond to his parent?
3. Abbey borrowed her best friend, Tasha’s, favorite hockey jersey to wear to a game. Abbey did not have Tasha’s permission to borrow the jersey. While Abbey was at the game, she accidentally made a big tear down the middle of the jersey. The next time she was with Tasha, Tasha was looking for her jersey. How is Abbey most likely to respond to Tasha?
4. Abbey borrowed her parent’s cellphone to bring with her while she was out with friends. Abbey did not have her parent’s permission to borrow the cellphone. While Abbey was with her friends, she accidentally dropped the phone in water and broke it. When she returned home, her parent was looking for their cellphone. How is Abbey most likely to respond to her parent?
5. Jeff was out at his friend’s house, but he forgot to watch the time and arrived home after his curfew. The next day his best friend, Terry, asked him where he was last night because they were supposed to study together. How is Jeff most likely to respond to his friend?
6. When Jeff arrived home past his curfew, his parent was already sleeping. The next morning, his parent asked him if everything was fine while he was at his friend’s house the other night. How is Jeff most likely to respond to his parent?
7. Kelly’s best friend, Sierra, invited her to watch her basketball team in a semi-finals game one evening after school. But Kelly didn’t feel like watching a basketball game. Sierra asks Kelly, “So, are you going to be there at the basketball game tonight?” How is Kelly most likely to respond to Sierra?
8. Kelly’s parent has been working for several months on painting an art piece. Kelly’s parent was able to sell their art piece to a very big art gallery downtown. Kelly’s parent invited Kelly to come see the painting on display on the opening night at the art gallery. But Kelly didn’t feel like going to an art gallery. Kelly’s parent asks Kelly “Are you ready to go to the art gallery?” How is Kelly most likely to respond to her parent?
9. There are tryouts for the school soccer team. Mitchell’s best friend, Terek, wants them both to try out for the team. Mitchell is not very good at soccer, but Terek is good at soccer. Terek asks Mitchell, “Are you going to try out for the team with me?” How is Mitchell most likely to respond to Terek?
10. Mitchell’s parent brings him to a new activity center to try some new activities. Mitchell tries one activity and is very clumsy at it, and he stops doing the activity and sits on a bench to wait for his parent to finish. Mitchell’s parent asks him “What are you doing sitting on the bench? Come on back!” How is Mitchell most likely to respond to his parent?
11. Kayla’s best friend Charlotte ends up trying out for the soccer team. But even though Charlotte is good at soccer, she doesn’t make the team. Charlotte is embarrassed about not making the team. One of Kayla and Charlotte’s classmates remembers that Charlotte was trying out for the soccer team and asks Kayla, “How did the soccer tryouts go for Charlotte?” How is Kayla most likely to respond to her classmate?
12. Kayla had told her parent that Charlotte was trying out for the soccer team. Charlotte was embarrassed that she didn’t make the soccer team. After school, Kayla’s parent asked her, “How did the soccer tryouts go for Charlotte?” How is Kayla most likely to respond to her parent?
